# Diabetes Island: Preliminary Impact of a Virtual World Self-Care Educational Intervention for African Americans With Type 2 Diabetes

**DOI:** 10.2196/games.3260

**Published:** 2014-08-08

**Authors:** Laurie Ruggiero, Ada Moadsiri, Lauretta T Quinn, Barth B Riley, Kirstie K Danielson, Colleen Monahan, Valerie A Bangs, Ben S Gerber

**Affiliations:** ^1^Institute for Health Research and Policy and Division of Community Health SciencesSchool of Public HealthUniversity of Illinois at ChicagoChicago, ILUnited States; ^2^College of NursingDepartment of Biobehavioral Health ScienceUniversity of Illinois at ChicagoChicago, ILUnited States; ^3^College of NursingDepartment of Health Systems ScienceUniversity of Illinois at ChicagoChicago, ILUnited States; ^4^Division of Transplant SurgeryDivision of Epidemiology & BiostatisticsUniversity of Illinois at ChicagoChicago, ILUnited States; ^5^Center for Advancement of Distance EducationSchool of Public HealthUniversity of Illinois at ChicagoChicago, ILUnited States; ^6^Jesse Brown VA Medical CenterChicago, ILUnited States; ^7^College of MedicineUniversity of Illinois at ChicagoChicago, ILUnited States

**Keywords:** minority group, type 2 diabetes mellitus, self-management

## Abstract

**Background:**

Diabetes is a serious worldwide public health challenge. The burden of diabetes, including prevalence and risk of complications, is greater for minorities, particularly African Americans. Internet-based immersive virtual worlds offer a unique opportunity to reach large and diverse populations with diabetes for self-management education and support.

**Objective:**

The objective of the study was to examine the acceptability, usage, and preliminary outcome of a virtual world intervention, Diabetes Island, in low-income African Americans with type 2 diabetes. The main hypotheses were that the intervention would: (1) be perceived as acceptable and useful; and (2) improve diabetes self-care (eg, behaviors and barriers) and self-care related outcomes, including glycemic control (A1C), body mass index (BMI), and psychosocial factors (ie, empowerment and distress) over six months.

**Methods:**

The evaluation of the intervention impact used a single-group repeated measures design, including three assessment time points: (1) baseline, (2) 3 month (mid intervention), and (3) 6 month (immediate post intervention). Participants were recruited from a university primary care clinic. A total of 41 participants enrolled in the 6 month intervention study. The intervention components included: (1) a study website for communication, feedback, and tracking; and (2) access to an immersive virtual world (Diabetes Island) through Second Life, where a variety of diabetes self-care education activities and resources were available. Outcome measures included A1C, BMI, self-care behaviors, barriers to adherence, eating habits, empowerment, and distress. In addition, acceptability and usage were examined. A series of mixed-effects analyses, with time as a single repeated measures factor, were performed to examine preliminary outcomes.

**Results:**

The intervention study sample (N=41) characteristics were: (1) mean age of 55 years, (2) 71% (29/41) female, (3) 100% (41/41) African American, and (4) 76% (31/41) reported annual incomes below US $20,000. Significant changes over time in the expected direction were observed for BMI (*P*<.02); diabetes-related distress (*P*<.02); global (*P*<.01) and dietary (*P*<.01) environmental barriers to self-care; one physical activity subscale (*P*<.04); and one dietary intake (*P*<.01) subscale. The participant feedback regarding the intervention (eg, ease of use, interest, and perceived impact) was consistently positive. The usage patterns showed that the majority of participants logged in regularly during the first two months, and around half logged in each week on average across the six month period.

**Conclusions:**

This study demonstrated promising initial results of an immersive virtual world approach to reaching underserved individuals with diabetes to deliver diabetes self-management education. This intervention model and method show promise and could be tailored for other populations. A large scale controlled trial is needed to further examine efficacy.

## Introduction

### The Burden of Diabetes

Diabetes is a serious worldwide public health challenge that has been growing in prevalence, with an estimated 347 million people with diabetes worldwide [1]. There are two major forms of diabetes, type 1 and 2, affecting 10% and 90% of the population, respectively [1]. The burden of diabetes, including prevalence and risk of complications, is greater for minorities, particularly African Americans [2]. Diabetes affects approximately 4.9 million, or 18.7% of African Americans 20 years or older [3]. African Americans are 1.8 times more likely to have diabetes, 2.6 to 5.6 times as likely to suffer from kidney disease, and 2.7 times as likely to suffer from lower-limb amputations as non-Hispanic whites [3].

### Harnessing the Virtual World

The Internet in general, and immersive virtual worlds, in particular, offer a unique opportunity to reach large and diverse populations with diabetes, while minimizing common barriers, such as lack of transportation or child care and scheduling conflicts. Immersive virtual worlds (eg, computer-based simulated 3-dimensional, 3-D, environments) are intended for users to inhabit and interact via an avatar, and communicate with others using voice and text chat tools. Second Life (SL) is one of the most frequently used virtual world environments, and offers a unique opportunity for reaching people with type 2 diabetes mellitus [4]. Several methodological and theoretical papers [5-7] have been published on the use of SL in supporting self-management in people with diabetes. To our knowledge, only one published study examined the outcomes of the use of a virtual environment for diabetes self-management education and support [8]. This pilot study demonstrated the feasibility and promising preliminary effects of this approach.

### African Americans and the Internet

National trends demonstrate increased Internet use among African Americans. For example, in 2010 African Americans were estimated to be the fastest growing population for in-home broadband Internet adoption [9]. In addition, the percentage of African American adults with a home broadband connection grew by 22% from 2009 to 2010 (46% in 2009; 56% in 2010; and 64% in 2013) [9,10].

### Study Purpose

The overall purpose of this study was to implement and evaluate the preliminary impact on self-care of a SL virtual world intervention (“Diabetes Island”) designed to provide diabetes self-management education to facilitate optimal diabetes self-management in low-income African Americans with diabetes attending primary care clinics. A secondary goal of this study was to purposefully recruit underserved individuals who might not have the opportunity to participate due to lack of computer/Internet skills and/or access. If effective, this intervention could be tailored for other groups and scaled up to reach large populations with diabetes.

This paper addresses the acceptability, usage, and preliminary impact of the Diabetes Island intervention on self-care and related outcomes. The main hypotheses were that the intervention would: (1) be perceived as acceptable and useful; and (2) improve diabetes self-care (eg, behaviors, barriers, and dietary intake) and self-care related outcomes, including glycemic control (A1C), body mass index (BMI), and psychosocial factors (ie, empowerment and distress) over six months. In addition, average weekly Diabetes Island log-ins will be provided to examine usage.

## Methods

### Study Design

The evaluation of the impact of the intervention involved a single-group repeated measures design with three intervention assessment time points: (1) baseline, (2) 3 month (mid intervention), and (3) 6 month (immediate post intervention).

### Setting, Eligibility Criteria, and Recruitment

The participants were recruited from a university-based primary care clinic. The eligible individuals were informed of the study details, invited to participate, and, those who were interested completed the consent process. Institutional Review Board (IRB) approval was obtained through the University of Illinois IRB.

The participants were enrolled in this study based on the following primary inclusion criteria: (1) African American; (2) age equal to or greater than 18 years; (3) fluent in English; (4) able to provide informed consent; (5) diagnosis of type 2 diabetes; (6) receiving medication therapy for diabetes (insulin or oral agents, or both); and (7) not pregnant, or planning a pregnancy during the study period.

### Run-In Phase

An initial run-in phase was conducted to serve multiple purposes, including to confirm that potentially eligible participants had basic skills in using the computer and SL; to ensure that individuals were committed to participation; and to gather feedback to further refine the intervention and support usage. Over approximately two months, clinic staff identified and referred 112 potential participants to the research team during routine clinic visits. A total of 109 participants were enrolled in this run-in phase. The run-in phase activities were held at the research site, and included study orientation, assessments, and trainings. A goal of the study was to include individuals with a wide range of computer and Internet skills at enrollment, including individuals with limited or without any computer and/or Internet skill. Therefore, to support this goal, an intensive 2 hour in-person training was conducted as part of the run-in phase to provide: (1) basic skills in the use of a laptop (eg, power on; use mouse; and use keyboard), (2) Internet (eg, open and close browser; use desktop icons; and use address box), and (3) SL (eg, log in; walk; view and keep objects; and use voice and text chat). The participants were able to attend the training a second time where needed or desired. Feedback was obtained from the participants during this process on the intervention, and observations were made regarding any challenges during use.

### Sample

A total of 69 participants were eligible for the intervention study (23/69 were lost to follow-up; 5 declined participation), and 41 consented and provided baseline assessments. There were four additional participants that withdrew prior to logging in at all; 37 participated in the intervention.

### Data Collection Procedure and Measures

#### Data Collection and Procedure

Assessment was conducted during the run-in period to collect basic self-report information on demographic, sociodemographic, and technology use characteristics of the participants. Self-report measures, BMI, and A1C were collected at baseline, 3 months (mid program), and 6 months (end of intervention) by trained research staff. The participants came to the Institute for Health Research and Policy at the University of Illinois at Chicago for training and each assessment. The self-report measures were administered using an interactive (touch screen) computerized assessment method that included audio through headsets (where desired). The participants received US $25 for completion of each assessment at the research site.

#### Strategies to Overcome Access Barriers

All of the participants in the intervention were provided with the same type of laptop to minimize barriers to access, and to ensure that everyone had a computer that would support the 3-D multimedia aspects of the program. Support for Internet access was also provided where needed to eliminate lack of Internet access as a barrier. The computers were set up by research staff to include only the software necessary for the study, and participants were instructed to use the computer only for study purposes during the study period. The participants were informed after the run-in period, and during the intervention study consent process, that they could retain the laptop at the completion of the study.

### Measures

#### Diabetes Control and Anthropometric Measures

Hemoglobin A1C level, a measure of long-term glycemic control, was obtained by a trained research staff member using the DCA2000+ Analyzer from Bayer (Mikashawa, IN). Height was measured using a portable stadiometer at baseline. Weight was measured using a Tanita Body Composition Analyzer at each assessment (Tanita Corporation of America, Inc).

#### Self-Care Measures

The self-care measures included: (1) the Summary of Diabetes Self-care Activities [11] (SDSCA) to assess diabetes self-care behavior; (2) the Environmental Barriers to Adherence Scale [12] (EBAS) to assess environmental barriers that interfere with self-care; and (3) the Fat-Related Diet Habits Questionnaire [13] (DHQ) to assess dietary intake.

The SDSCA was modified to include all of the original items, plus additional items to assess walking and specific nutritional content areas emphasized in the program (ie, reading food labels, choosing foods from the five food groups, and drinking water). The original SDSCA subscales were used for glucose self-testing, foot care, and medication use. The revised SDSCA subscales were examined in this study for general diet, specific diet, and physical activity. The medication use scale has been found to display a ceiling effect [11]; therefore, medication adherence was excluded as a self-care outcome in the current study. We additionally computed a composite score across these subscales, as done in previous studies [14].

The EBAS [12] examines 60 environmental barriers across four areas of self-care (ie, diet, exercise, blood glucose testing, and medication). A global barriers score is calculated by summing the responses to all of the items, and subscales are calculated for each of the self-care behavior areas. The DHQ [13] is a 22 item scale that assesses eating habits, and provides five factor scores measuring low-fat/nonfat substitution, modification of meat intake, avoidance of frying, fruit and vegetable replacement, and avoidance of fat. Lower scores over time, on the DHQ, reflect an increase in healthy eating.

#### Psychosocial Measures

The psychosocial measures included: (1) the Diabetes Empowerment Scale-Short Form [15] used to assess psychosocial self-efficacy and empowerment; and (2) the Diabetes Distress Scale [16,17] to assess diabetes related problems and distress.

#### Acceptability and Usage

Questions were included in the 6 month assessment to examine acceptability of the intervention. Acceptability questions covered topics related to ease of use, interest and comfort level, and perceived personal impact. Log-in data for Diabetes Island were collected to examine usage.

### Intervention Components

#### Overview and Development

The participants in the intervention study (n=37) were given access to the intervention components during a 6 month period. The intervention components included a study website for communication and tracking, as well as access to a SL dedicated study space, Diabetes Island, where a variety of educational activities were available as described below. The intervention was designed and developed by a multidisciplinary (physician, nurse educator, psychologist, and dietitian) research team, including experts in content, SL, and behavior change; and a technology development team, including designers and programmers. Input was also obtained from two advisory committees: (1) one included African American individuals with diabetes, and (2) the other included health educators who implement community-based diabetes education programs within similar populations. Advisory committee meetings were conducted to obtain input regarding the overall design of the virtual world intervention, and the specific content areas of the educational components. The development of the educational component was informed through input from the multidisciplinary research team, and feedback from advisory committees regarding patient needs. In addition, behavior change theory, particularly social cognitive theory [18], was used to guide the development of the intervention. The emphasis was on increasing knowledge (eg, formal and informal presentations; educational resources available); building self-efficacy through in-world contextual activities (eg, successfully making healthier food choices in a programmed fast food activity with feedback); observational learning or learning from other participants (eg, in-world interactive discussions on healthier eating); reinforcement (eg, receiving points for participation in educational activities); and goal setting (eg, setting personal activity goals using website). In addition, observations and feedback from the participants during the run-in phase were used to modify the intervention and its implementation. For example, the participants had challenges with using chat features, navigation, and working with objects. Therefore, in-world technical support sessions were added and modifications (eg, instructional signage) were made to the island to help the participants with various aspects of using SL (eg, navigation, keeping/using objects, and chatting).

#### Study Website

A study website was developed to: (1) communicate with the participants about project activities, events, and announcements; (2) track individual goals, activities, and accomplishments; (3) provide individualized feedback on activity completion; and (4) provide study and technical support information.

#### Second Life Intervention, Diabetes Island

SL is a virtual world or computer-based simulated 3-D environment intended for users to inhabit and interact via an avatar (a virtual world representation of the user). Users are identified by their avatar name, and can communicate with other users through voice and text chat tools. Real life anonymity is guaranteed unless the user chooses to share his or her real-world identity. Our study island was password protected, and only the study participants and the project team were provided with access to Diabetes Island.

Using their avatars, the participants could access a variety of educational resources available 24/7 (ie, on-demand) at different locations on Diabetes Island. In particular, these included several programmed scenarios (described below); nutrition labels and educational signage around the island; and a learning center with a variety of written materials (eg, National Diabetes Education Program materials) and videos (eg, American Diabetes Association videos). There were four programmed scenarios that provided interactive contextual learning opportunities particularly related to healthy eating and physical activity. For example, “choosing a healthy lunch on the go” focused on making healthier choices through reading food labels at a fast food restaurant.

**Figure 1:**
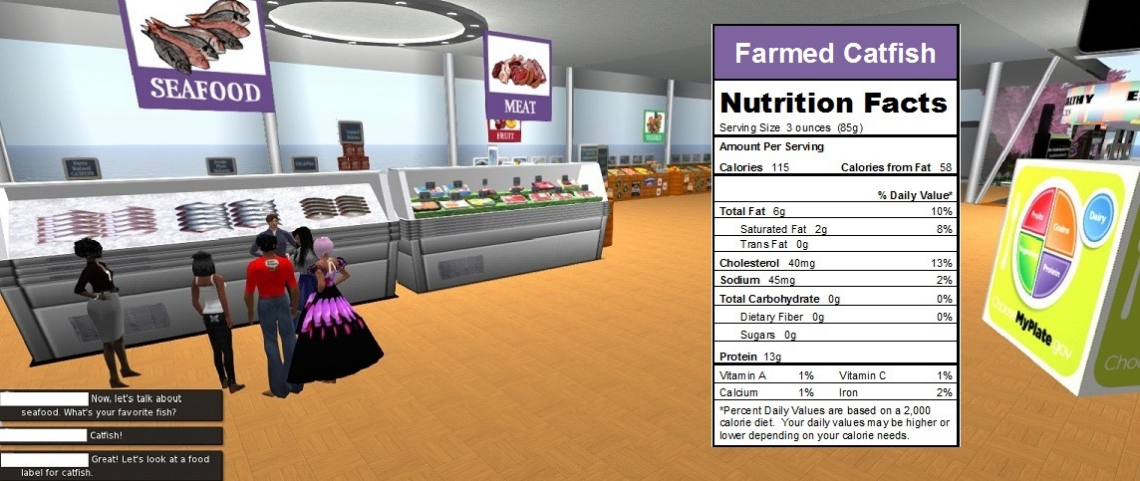
Dietitian-led discussion of reading food labels in the grocery store.

Health professionals (as avatars) provided real time formal and informal educational sessions. A series of 10 formal presentation sessions (30-60 minutes in length) were offered, including: (1) six healthy eating topics led by a registered dietitian; (2) one on medication adherence led by a pharmacist; (3) one on physical activity led by an exercise researcher; and (4) two led by a nurse Certified Diabetes Educator on the “ABC’s” of diabetes and proper foot care. Several dietitian-facilitated real time discussions on healthier eating were offered in the grocery store, fast food restaurant, and home. An example of a dietitian-facilitated discussion opportunity involved meeting at the grocery store to discuss making healthier choices while shopping and reading food labels (Figure 1 shows this image). The participants received points for engaging in various Diabetes Island educational activities, and these points could be traded for clothing for their avatars.

Social activities, such as dance parties and sporting/leisure activities, were regularly offered on Diabetes Island to encourage the participants to visit frequently, and, thereby, facilitate engagement in the intervention. In-world technical support sessions and walking tours of the island were offered periodically to help the participants learn about the island and its use. When participants needed or requested guidance or information regarding their medical care, the project staff and presenters referred them to their primary care providers.

#### Intervention Procedure

The participants were given access to Diabetes Island for a 6 month period. There were regular (generally weekly) professional-led virtual world educational events, and periodic virtual world leisure/social events as described earlier. In addition, the participants had access to Diabetes Island 24 hours per day, seven days a week, and could access educational resources and participate in programmed activities on their own schedule. Since the participants’ computer, Internet, and SL skills were diverse, the research team also provided periodic in-world technical support sessions to answer questions and assist with common technical challenges (eg, chatting, keeping/interacting with SL objects, navigation, and downloading updates). Instructional/technical support was also available through written manuals on the study website, and on an individual basis by telephone when in-world support sessions were not sufficient.

### Analyses

In order to take into consideration the correlated nature of longitudinal data, and the fact that observations over time are nested within individuals, we performed a series of mixed-effects analyses with time as a single repeated measures factor. This approach is also quite compatible with an intent-to-treat approach to data analysis, as it does not require that respondents have complete data at all waves to be included in the analysis. The present analyses employed a Toeplitz covariance structure for the repeated observations, which assumes that the correlation between observations decreases the further out observations are in time. If a significant time effect was observed, subsequent pairwise post-hoc analyses were performed using the Sidak test. All analyses were performed using SPSS version 21.0 (IBM Corporation).

## Results

### Overview

The results section will describe characteristics of the study sample and the comparisons across time for the clinical, self-care, and psychosocial variables.

### Baseline Sociodemographic Characteristics of Sample

The characteristics of the intervention sample (N=41) can be seen in Table 1. This group included individuals with the following characteristics: (1) mean age of 55 years; (2) 71% (29/41) female; (3) 76% (31/41) had a high school education or higher; (4) 22% (9/41) reported annual incomes above US $20,000; (5) 12% (5/41) reported current employment; (6) 98% (40/41) had insurance coverage, including Medicare/Medicaid; and (7) 12% (5/41) were married.

**Table 1:** Characteristics of the study participants (N=41).

Variables		Mean (SD) or n (%)^a^
Age		55.2 (9.6)
Female		29 (71)
**Education**		
	< High school	10 (24)
	High school graduate	12 (29)
	> High school	19 (47)
**Annual household income**		
	Less than US $20,000	31 (76)
	More than US $20,000	9 (22)
	Don’t know/refused	1 (2)
**Employment status** ^b^		
	Currently employed	5 (12)
	Unemployed	1 (3)
	Other	35 (85)
**Health insurance** ^c^		
	Yes	40 (98)
	No	1 (2)
**Marital status**		
	Married	5 (12)
	Never married	17 (42)
	Other^d^	19 (46)

^a^A subgroup of 41 individuals enrolled in the intervention study, 4 withdrew prior to initiating the intervention.

^b^The employment status category of “other” includes homemakers, students, and those retired and/or unable to work.

^c^The health insurance status category of “yes” includes Medicare and Medicaid.

^d^The marital status category of “other” includes separated, divorced, and widowed persons.

### Baseline Technology-Related Characteristics

Table 2 shows the baseline technology characteristics of the study participants. Prior to participation, only 46% (19/41) of the participants had Internet access at home; 76% (31/41) had ever used video or computer games; 29% (12/41) reported experience with virtual or 3-D worlds previously (only one with SL); and 54% (22/41) reported that household members play video or computer games. There was diversity in the range of self-rated computer skills with 37% (15/41) poor-very poor, 34% (14/41) fair, and 29% (12/41) good-very good. The participants’ reported baseline computer/Internet skills and access indicates achievement of our recruitment goal of reaching diverse individuals, including a subgroup that might not otherwise have the opportunity to participate due to lack of resources and/or basic computer/Internet skills.

**Table 2:** Technology characteristics of the study participants (N=41).

Variables		n (%)
Home Internet		19 (46)
**Use of video or computer games**		
	Never	10 (24)
	Used to, but do not anymore	9 (22)
	Currently do, sometimes	15 (37)
	Currently do, frequently	7 (17)
Experience with virtual or 3-D world		12 (29)
**Self-rated computer skills**		
	Very poor-poor	15 (37)
	Fair	14 (34)
	Good-very good	12 (29)
Household member actively plays video or computer games		22 (54)

### Acceptability Results

Table 3 is a summary of the feedback on Diabetes Island provided immediately post intervention (ie, 6 months). The feedback regarding acceptability, ease of use of Diabetes Island, and perceived impact was consistently positive.

**Table 3:** Results of acceptability survey (n=33).

Acceptability item	Strongly agree, n (%)	Agree, n (%)	Disagree/ strongly disagree, n (%)
Diabetes Island is interesting.	23 (70)	9 (27)	1 (3)
Diabetes Island was easy to get around.	12 (36)	14 (43)	7 (21)
Diabetes Island is a comfortable place to spend time.	17 (52)	14 (42)	2 (6)
Diabetes Island motivated me to take better care of my diabetes.	15 (45)	16 (49)	2 (6)
Diabetes Island would be helpful to other people like me.	23 (70)	9 (27)	1 (3)
I learned things through Diabetes Island that I can apply to my own life.	22 (67)	11 (33)	0 (0)
I would recommend Diabetes Island to a friend or family member with diabetes.	22 (67)	10 (30)	1 (3)
I have used the things I learned through Diabetes Island to take better care of my diabetes.	16 (49)	17 (51)	0 (0)

### Diabetes Island Usage

**Figure 2:**
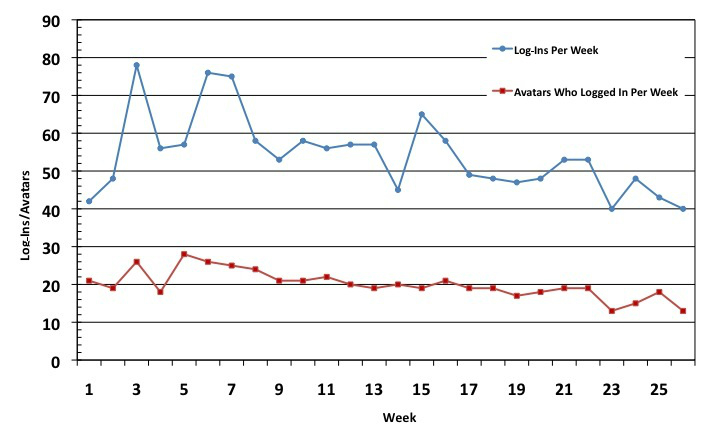
Total weekly log-ins and total avatars that logged in each week.

Although there was a sharp spike in log-ins for the first seven weeks, and a sharp decrease in the last four weeks, the rates were relatively consistent for much of the intervention period (Figure 2 shows this information). The total number of log-ins for the first half of the program was 771 compared to 637 for the second half. The total log-ins per week ranged from 40 to 78, with an average weekly log-in rate of 55. The average number of avatars that logged in per week was 20, with a range of 13 to 28. Consistent with the log-in rates, the usage patterns per avatar were greater in the first half of the time period, with ranges of 49% (18/37) to 76% (28/37) of avatars logging in each week compared with 35% (13/37) to 57% (21/37) in the second half.

### Comparisons Across Time for Glycemic Control, Body Mass Index, Self-care, and Psychosocial Variables

Table 4 summarizes the results for A1C, BMI, self-reported self-care patterns, and self-care psychosocial variables across time. Significant changes over time were observed for BMI, self-care behaviors, environmental barriers to self-care, and diabetes related distress. In addition, a trend (*P*=.053) was observed for A1C, with decreases occurring from 3 to 6 months. The BMI level demonstrated a significant, but modest decrease across time. For the self-care behavior measure, significant improvements across time were found on the summary score and revised physical activity scale. There was one subscale on the DHQ (modify meat) that showed significant improvement, and a trend was identified on another subscale (substitution). The environmental barriers global score and diet specific subscale significantly improved across time. Scores on the diabetes distress scale also improved significantly across time. No difference was found on the empowerment scores across time. Among significant outcomes, post-hoc analyses revealed that significant changes occurred from baseline to 3 months. However, significant changes from 3 to 6 months were observed on diabetes related distress, and baseline to 6 months for BMI, diabetes distress, and the “modify meat” subscale of the DHQ.

The revised scores for the diabetes self-care measure include items added to reflect specific content of the program, such as reading food labels and walking.

**Table 4:** A1C, BMI, self-care, and psychosocial measures across time, baseline through 6 months.

Variables		Baseline (N=41), mean (SE)	3 months (n=36), mean (SE)	6 months (n=36), mean (SE)	*P*
A1C		7.4 (0.21)	7.4 (0.22)	7.1 (0.22)	.05
BMI		39.3 (1.60)	38.8 (1.66)	38.6 (1.66)	.02^c^
**Diabetes self-care behavior**					
	Summary score	5.1 (0.15)	5.5 (0.16)	5.4 (0.16)	.03^c^
	General diet	5.6 (0.26)	6.0 (0.27)	6.0 (0.27)	.22
	General diet-rev	5.4 (0.25)	5.9 (0.26)	5.9 (0.26)	.09
	Specific diet	4.8 (0.17)	5.0 (0.18)	4.8 (0.18)	.42
	Specific diet-rev	5.6 (0.15)	5.7 (0.16)	5.6 (0.16)	.91
	Exercise	4.0 (0.32)	4.7 (0.33)	4.5 (0.34)	.12
	Physical activity-rev	3.9 (0.31)	4.7 (0.33)^a^	4.5 (0.33)	.04^c^
	Self-testing	6.6 (0.28)	6.9 (0.29)	6.6 (0.29)	.46
	Foot care	6.0 (0.33)	6.0 (0.35)	6.4 (0.35)	.29
**Diabetes health questionnaire**					
	Substitution	2.7 (0.13)	2.8 (0.13)	2.5 (0.13)	.05
	Avoid frying	1.9 (0.08)	1.8 (0.08)	1.8 (0.09)	.18
	Replacement	2.8 (0.11)	2.7 (0.12)	2.7 (0.12)	.43
	Modify meat	2.6 (0.13)	2.4 (0.13)	2.2 (0.14)^b^	.01^c^
	Avoid fat	3.0 (0.07)	2.9 (0.08)	3.0 (0.08)	.70
**Environmental barriers to adherence**					
	Global score	2.2 (0.11)	1.9 (0.11)^a^	2.0 (0.11)	.01^d^
	Diet subscale	2.6 (0.12)	2.2 (0.13)^a^	2.3 (0.13)	.01^d^
	Exercise subscale	2.5 (0.12)	2.3 (0.13)	2.3 (0.13)	.17
	Glucose testing	1.9 (0.14)	1.6 (0.14)	1.7 (0.14)	.14
	Medication subscale	1.8 (0.14)	1.7 (0.14)	1.5 (0.14)	.19
Diabetes empowerment		4.1 (0.12)	4.2 (0.13)	4.2 (0.13)	.89
Diabetes distress scale		2.4 (0.15)	2.3 (0.15)	1.9 (1.60)^a,b^	.02^c^

^a^significant, *P*<.05, post-hoc pairwise difference between the current and previous time point

^b^significant, *P*<.05, post-hoc pairwise difference between 6 months and baseline

^c^ *P*<.05 for mixed-effects model analyses including baseline, 3 month, and 6 month time points

^d^ *P*<.01 for mixed-effects model analyses including baseline, 3 month, and 6 month time points

## Discussion

### Diabetes Island Intervention

This paper described the acceptability, usage, and preliminary impact of Diabetes Island with low-income African Americans with type 2 diabetes attending a primary care clinic. The results of this study support our main hypotheses. The Diabetes Island intervention demonstrated participant acceptability, regular usage, and promising outcomes, including improvements in BMI, diabetes related distress, physical activity, dietary intake, and environmental barriers to self-care in a group of low-income African American adults attending a primary care clinic.

As planned, the group represented a diverse group regarding Internet/computer skills and Internet access prior to study participation. At baseline, less than half had Internet at home, and greater than two-thirds rated their computer skills as very poor to fair. The characteristics of the sample are generally consistent with those of individuals who are less likely to access Internet-based health information, especially those who are older and with lower education levels and lower income [19]. It is encouraging that once the participants were provided with the basic skills and resources to access the virtual world intervention, the majority logged in regularly during the first two months, and around half logged in each week on average across the six months.

The responses from the participants regarding the acceptability and usefulness of Diabetes Island were consistently positive. The participants appeared to be engaged in Diabetes Island, and reported that they found it useful to them and felt it would be useful to others like them. Therefore, once the access barriers are removed (eg, Internet/computer skills and access), Internet-based approaches might provide an additional strategy to reach underserved populations. In particular, African Americans represent a group that is adopting broadband Internet use at rates greater than other ethnic groups [9,10]. Second, the majority of our sample was not working outside the home due to retirement, lack of a job, being a homemaker, and/or disability. Being able to access interventions from home through the Internet would be a useful way to reach this population and overcome common barriers, such as scheduling, lack of time, and transportation. In addition, this approach offers an opportunity to interact with others with diabetes in a way that is anonymous and convenient.

The intervention outcome findings demonstrate positive changes in motivation and self-care. Self-care patterns were consistent with the literature, with the lowest levels found for lifestyle activities (ie, eating and physical activity habits), and higher levels for discrete behaviors (eg, medication use and foot care) [20]. In addition, the level of self-care increased across time in general, and was significantly improved for one physical activity and one healthy eating subscale. The intervention also showed promise in impacting A1C and BMI. The participants also indicated a reduction in their overall environmental barriers to self-care adherence, specifically related to eating behaviors. It is notable that changes related to healthy eating were the most consistent positive changes found in this study. This is an expected finding since there was a greater focus on topics related to healthy eating in virtual world professional-led educational events and programmed activities.

The examination of psychosocial variables showed significant reductions in diabetes related distress, but no significant changes in empowerment. The changes in diabetes distress are consistent with those found in our work with a similar population [21], and support the positive impact of the Diabetes Island intervention. Lack of change in empowerment was unexpected, and should be further examined in future research.

It is important to note that removing barriers to accessing the intervention was a goal of the study. To address this goal, basic computer/Internet skills and technology resources/support were provided to facilitate participation in the intervention. Therefore, for sustainability, this intervention would need to be delivered within a context where individuals currently have or can be provided with the necessary skills and resources to utilize this intervention. Innovative models need to be examined to disseminate this intervention outside of a research study. A potential translation model that may help overcome this resource challenge is to offer this intervention through community organizations and/or health centers that have available technology centers and that also ideally offer basic training in computer and Internet use.

### Limitations and Directions for Future Research

Although this study provides promising preliminary findings regarding acceptability, usage, and outcomes of this intervention approach, there are several limitations to consider. In particular, the small sample size, quasi-experimental design, and sample selection are limitations. The small sample size may have limited the power to detect differences on some variables, and the lack of a comparison group necessitates cautious interpretation of the findings pending further research. Although a goal of the study was to focus on low-income individuals with a range of Internet and computer skills in order to bridge the digital divide, this goal served as a limitation to the generalizability of the findings. Future research is needed that includes a large diverse sample and comparison group, ideally using a randomized design. Since extensive training was provided in the run-in phase, and ongoing technical support was provided throughout the study to facilitate participation, the feasibility of this intervention outside of a research study needs further investigation. In addition, the intervention included a variety of components, and the participants may have engaged in some components and not others. Therefore, it is not possible to determine the most important components of the intervention from this study. Further research is needed to more carefully examine what components of this intervention impact different outcome measures, and how effective components can be delivered most efficiently with least cost.

### Conclusions

This study demonstrated promising initial results of an immersive virtual world approach to reaching underserved individuals with diabetes to deliver diabetes self-management education. The study implementation bridged the digital divide to allow those traditionally lacking the necessary resources and skills to participate. This intervention model and method shows promise, and could be tailored for other groups. The findings address the primary questions of this paper, but raise new questions about this intervention approach and ways it might be tailored for different groups, and implemented using different models to reach those without technology/Internet access and skills. A large scale controlled trial is needed to further examine efficacy.

## Abbreviations

3-D: 3-dimensional
A1C: glycemic control
BMI: body mass index
DHQ: Diet Habits Questionnaire
EBAS: Environmental Barriers to Adherence Scale
IRB: Institutional Review Board
SDSCA: Summary of Diabetes Self-care Activities
SL: Second Life
